# Supplementary material for: DeMAG predicts the effects of variants in clinically actionable genes by integrating structural and evolutionary epistatic features
Source: Nat Commun. 2023 Apr 19;14:2230. doi: 10.1038/s41467-023-37661-z (PMC10115847; doi:10.1038/s41467-023-37661-z)
Supplement: Supplementary file 1 — Supplementary Information [file 41467_2023_37661_MOESM1_ESM.pdf]

Supplementary Information

DeMAG predicts the effects of variants in clinically actionable genes by integrating structural and evolutionary epistatic features

Authors:

Federica Luppino[1,2], Ivan A. Adzhubei[3,4], Christopher A. Cassa[3*], Agnes Toth-Petroczy[1,2,5*]

Affiliations:

1: Max Planck Institute of Molecular Cell Biology and Genetics, Dresden 01307, Germany
2: Center for Systems Biology Dresden, Dresden 01307, Germany
3: Brigham and Women's Hospital Division of Genetics, Harvard Medical School, Boston, MA, 02115 USA
4: Department of Biomedical Informatics, Harvard Medical School, Boston, MA 02115 USA
5:Cluster of Excellence Physics of Life, TU Dresden, 01062 Dresden, Germany

*Correspondence: ccassa@bwh.harvard.edu and toth-petroczy@mpi-cbg.de

List of Supplementary Figures and Supplementary Tables:

Supplementary Figure 1. ClinVar pathogenic annotation is biased for actionable genes.

Supplementary Figure 2. DeMAG training set reaches high balance between the pathogenic and benign class both between and within genes.

Supplementary Figure 3. AlphaFold2 structure predictions cover 100% of positions in the 59 ACMG SF genes.

Supplementary Figure 4. Most ClinVar VUSs are predicted as functional variants by DMS data.

Supplementary Figure 5. Most HGMD variants have a ClinVar label.

Supplementary Figure 6. Spatially close and co-evolving residues are enriched in the same phenotypic effect.

Supplementary Figure 7. Partners score is significantly higher in Pfam domains and in ordered regions.

Supplementary Figure 8. Epistatic and structural features increase sensitivity for "pathogenic" genes and specificity for "benign" genes.

Supplementary Figure 9. Most genes benefit from the epistatic and structural features.

Supplementary Figure 10. The ClinVar test set contains 853 pathogenic and 433 benign variants.

Supplementary Figure 11. DeMAG correctly identifies pathogenic and benign regions (coldspot) in BRCA1.

Supplementary Figure 12. Abundant, high-quality and well-balanced data only available for few ClinVar genes.

Supplementary Figure 13. EVmutation covers 74% of ACMG SF sites and 23% of residues are disordered.

Supplementary Figure 14. Bootstrap confidence intervals for ClinVar testing set.

Supplementary Figure 15. Bootstrap confidence intervals for the Estonian variants testing set.

Supplementary Table 1. DeMAG model is balanced among different statistics metrics.

Supplementary Table 2. DeMAG features.

Supplementary Table 3. DeMAG (ClinVar) model is balanced among different statistics metrics.

Supplementary Table 4. DeMAG generalizes to additional 257 disease associated genes.

Supplementary Table 5. DeMAG training set data sources.

Supplementary Figures

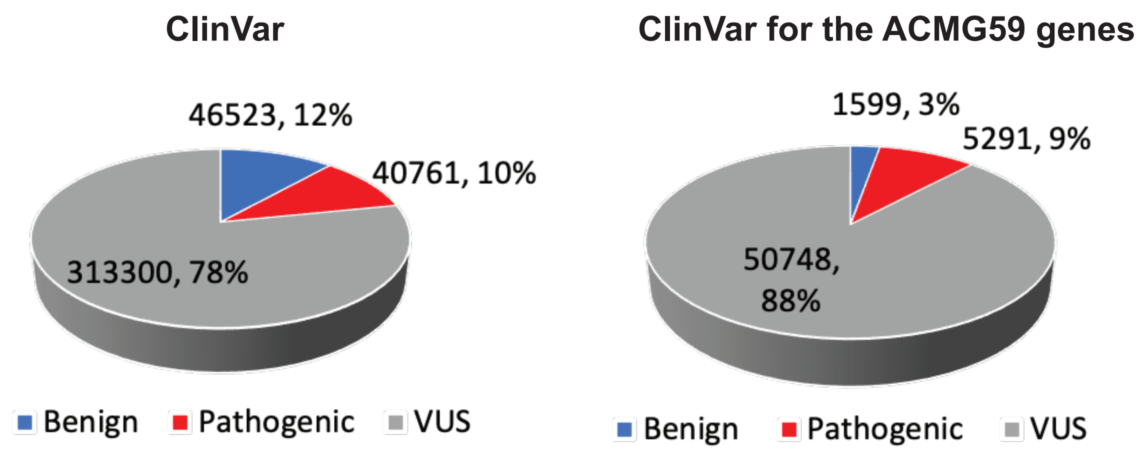

Supplementary Figure 1. ClinVar pathogenic annotation is biased for actionable genes.

Clinical annotation of ClinVar[1] variants for all available genes (left) and for the 59 ACMG SF v2.0 genes[2] (right). The proportion of Variants of Uncertain Significance (VUSs) is high overall. The ratio between pathogenic and benign variants is balanced for all genes (12% and 10%) and biased towards the pathogenic class (9% vs. 3% benign variants) for the ACMG SF genes.

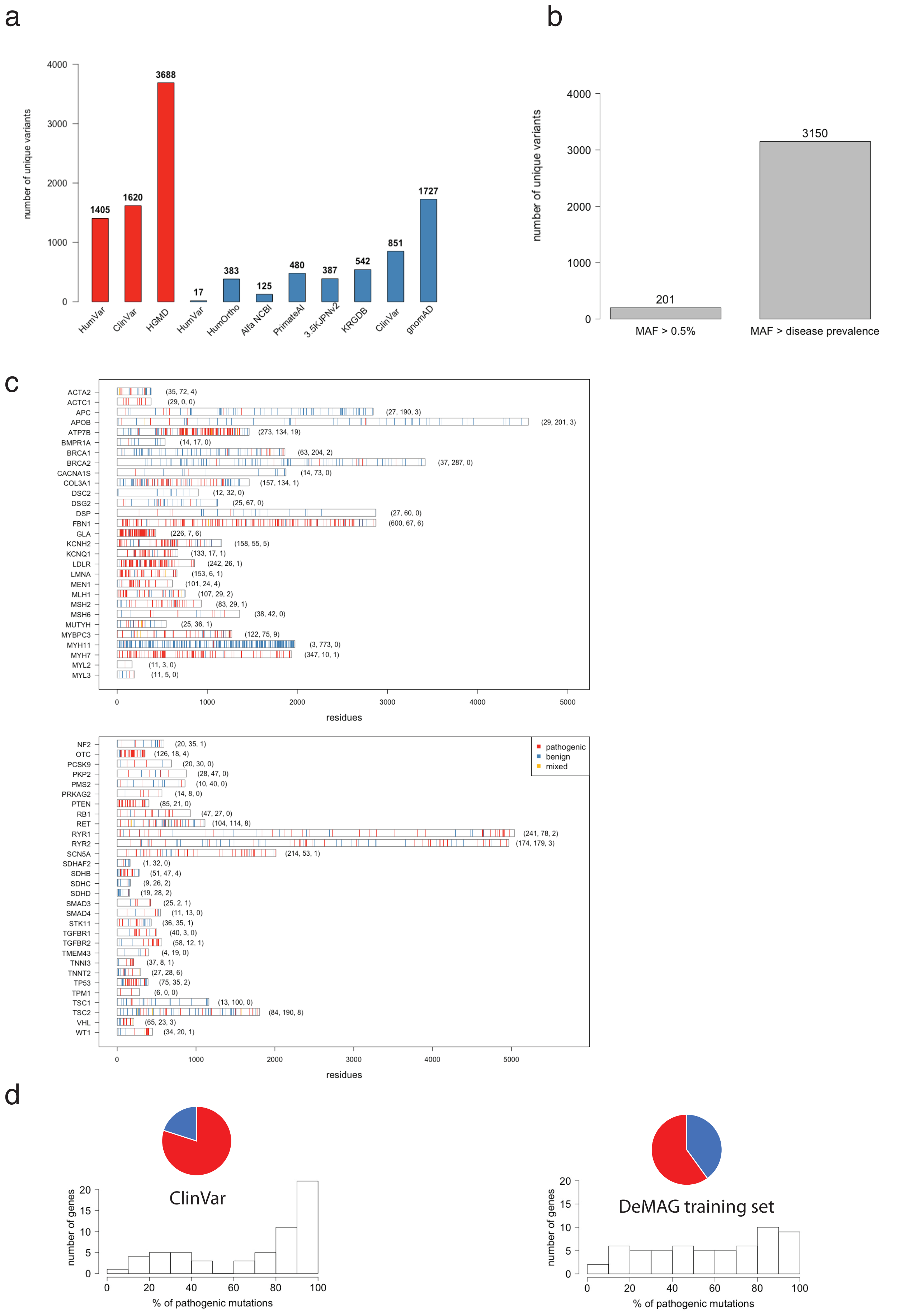

a
number of unique variants
4000
3000
2000
1000
0
1405
1620
3688
17
383
125
480
387
542
851
1727
HumVar
ClinVar
HGMD
HumVar
HumOrtho
Alfa NCBI
PrimateAI
3.5KJPNv2
KRGDB
ClinVar
gnomAD
b
number of unique variants
201
3150
MAF > 0.5%
MAF > disease prevalence
c
ACTA2 (35, 72, 4)
ACTC1 (29, 0, 0)
APC (27, 190, 3)
APOB (29, 201, 3)
ATP7B (273, 134, 19)
BMPR1A (14, 17, 0)
BRCA1 (63, 204, 2)
BRCA2 (37, 287, 0)
CACNA1S (14, 73, 0)
COL3A1 (157, 134, 1)
DSC2 (12, 32, 0)
DSG2 (25, 67, 0)
DSP (27, 60, 0)
FBN1 (600, 67, 6)
GLA (226, 7, 6)
KCNH2 (158, 55, 5)
KCNQ1 (133, 17, 1)
LDLR (242, 26, 1)
LMNA (153, 6, 1)
MEN1 (101, 24, 4)
MLH1 (107, 29, 2)
MSH2 (83, 29, 1)
MSH6 (38, 42, 0)
MUTYH (25, 36, 1)
MYBPC3 (122, 75, 9)
MYH11 (3, 773, 0)
MYH7 (347, 10, 1)
MYL2 (11, 3, 0)
MYL3 (11, 5, 0)
0
1000
2000
3000
4000
5000
residues
NF2 (20, 35, 1)
OTC (126, 18, 4)
PCSK9 (20, 30, 0)
PKP2 (28, 47, 0)
PMS2 (10, 40, 0)
PRKAG2 (14, 8, 0)
PTEN (85, 21, 0)
RB1 (47, 27, 0)
RET (104, 114, 8)
RYR1 (241, 78, 2)
RYR2 (174, 179, 3)
SCN5A (214, 53, 1)
SDHAF2 (1, 32, 0)
SDHB (51, 47, 4)
SDHC (9, 26, 2)
SDHD (19, 28, 2)
SMAD3 (25, 2, 1)
SMAD4 (11, 13, 0)
STK11 (36, 35, 1)
TGFBR1 (40, 3, 0)
TGFBR2 (58, 12, 1)
TMEM43 (4, 19, 0)
TNNI3 (37, 8, 1)
TNNT2 (27, 28, 6)
TP53 (75, 35, 2)
TPM1 (6, 0, 0)
TSC1 (13, 100, 0)
TSC2 (84, 190, 8)
VHL (65, 23, 3)
WT1 (34, 20, 1)
pathogenic
benign
mixed
0
1000
2000
3000
4000
5000
residues
d
ClinVar
number of genes
20
15
10
5
0
0
20
40
60
80
100
% of pathogenic mutations
DeMAG training set
number of genes
% of pathogenic mutations

Supplementary Figure 2. DeMAG training set reaches high balance between the pathogenic and benign class both between and within genes.

a) The proportion of unique benign (blue) and pathogenic (red) variants in our training set from the different source databases. See Methods for a detailed description of the sources.
b) The number of unique common variants from the population databases defined based on two strategies for thresholding the minor allele frequency (MAF): 201 variants satisfy MAF>0.5%[3], and 3150 variants if MAF greater than the specific disease prevalence[4,5] (see Supplementary Data 1).
c) Pathogenic and benign variants per genes. The x-axis indicates the position in the protein sequence and the y-axis indicates the gene name. The values in parenthesis indicate the number of pathogenic, benign and mixed variants, respectively. A mixed variant identifies a residue position associated both with benign and pathogenic mutations in the training set.
d) The pie chart on the left indicates the annotation of variants for the ACMG SF genes in the ClinVar database: 20% benign and 80% pathogenic variants. The pie chart on the right shows the annotation of variants for DeMAG training set: 60% are pathogenic (red) and 40% are benign (blue) variants. The histograms show the distribution of pathogenic variants (%) within genes. On the left, almost 65% of genes have more than 70% of pathogenic variants in the ClinVar database. In our training set, only 40% of genes have more than 70% pathogenic variants.

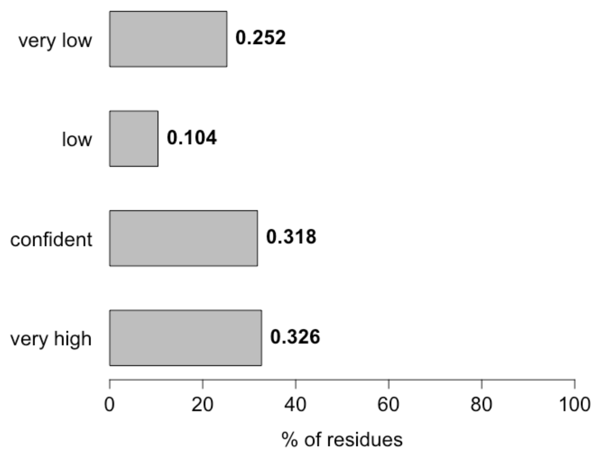

Supplementary Figure 3. AlphaFold2 structure predictions cover 100% of positions in the 59 ACMG SF genes.

AlphaFold2 confidence metric pLDDT score for each residue in the ACMG SF genes. AlphaFold2 structure predictions cover 100% of residues. However, 36% of the residues are predicted with low and very low confidence.

	Review Stars			
**Clinical Significance**	**0**	**1**	**2**	**3**
Benign	0	3	6	15
Pathogenic	1	19	41	30
VUS	1164	202	142	1
Conflicting Pathogenicity	1	62	0	1

VEPs	Sensitivity	Specificity	Accuracy	MCC	AUC	Variants Predicted (n = 1587)	VEP's coverage
DeMAG	0.92	0.51	0.6	0.35	0.84		1
SIFT4G	1	0.02	0.22	0.06	0.76	1587	1
DeMAG	0.92	0.51	0.6	0.35	0.84		1
REVEL	0.98	0.18	0.34	0.19	0.87	1587	1
DeMAG	0.95	0.54	0.62	0.39	0.88		1
DEOGEN2	0	1	0.8	0	0.86	1109	0.7
DeMAG	0.95	0.54	0.62	0.39	0.88		1
PolyPhen2	0.46	0.77	0.7	0.2	0.67	1109	0.7
DeMAG	0.92	0.51	0.6	0.35	0.84		1
VEST4	0.96	0.41	0.52	0.31	0.88	1587	1
DeMAG	0.92	0.51	0.59	0.35	0.84		1
M-CAP	1	0	0.2	0.01	0.86	1574	0.99
DeMAG	0.94	0.41	0.55	0.33	0.82		1
EVE	0.99	0.23	0.42[1]	0.25	0.82	1213	0.76

[1]The accuracy is 44% if misclassified variants based on EVE's Uncertain class are included.

	Review Stars			
**Clinical Significance**	**0**	**1**	**2**	**3**
Pathogenic	0	1	2	1
VUS	1	8	5	0
Conflicting Pathogenicity	0	1	0	0
Not annotated in ClinVar		45		

VEPs	Sensitivity	Specificity	Accuracy	MCC	AUC	Variants Predicted (n = 53)	VEP's coverage
DeMAG	1	0.18	0.47	0.27	0.7		1
SIFT4G	1	0	0.36	0	0.56	53	1
DeMAG	1	0.18	0.47	0.27	0.7		1
REVEL	0.95	0.21	0.47	0.21	0.72	53	1
DeMAG	1	0.18	0.47	0.27	0.7		1
DEOGEN2	1	0.06	0.4	0.15	0.81	53	1
DeMAG	1	0.18	0.47	0.27	0.7		1
PolyPhen2	0.79	0.29	0.47	0.09	0.5	53	1
DeMAG	1	0.18	0.47	0.27	0.7		1
VEST4	1	0.09	0.42	0.18	0.72	53	1
DeMAG	1	0.18	0.47	0.27	0.7		1
M-CAP	1	0	0.36	0	0.53	53	1
DeMAG	1	0.15	0.48	0.25	0.71		1
EVE	0.25	0.69	0.52[1]	-0.06	0.56	42	0.79

[1]The accuracy is 44% if misclassified variants based on EVE's Uncertain class are included.

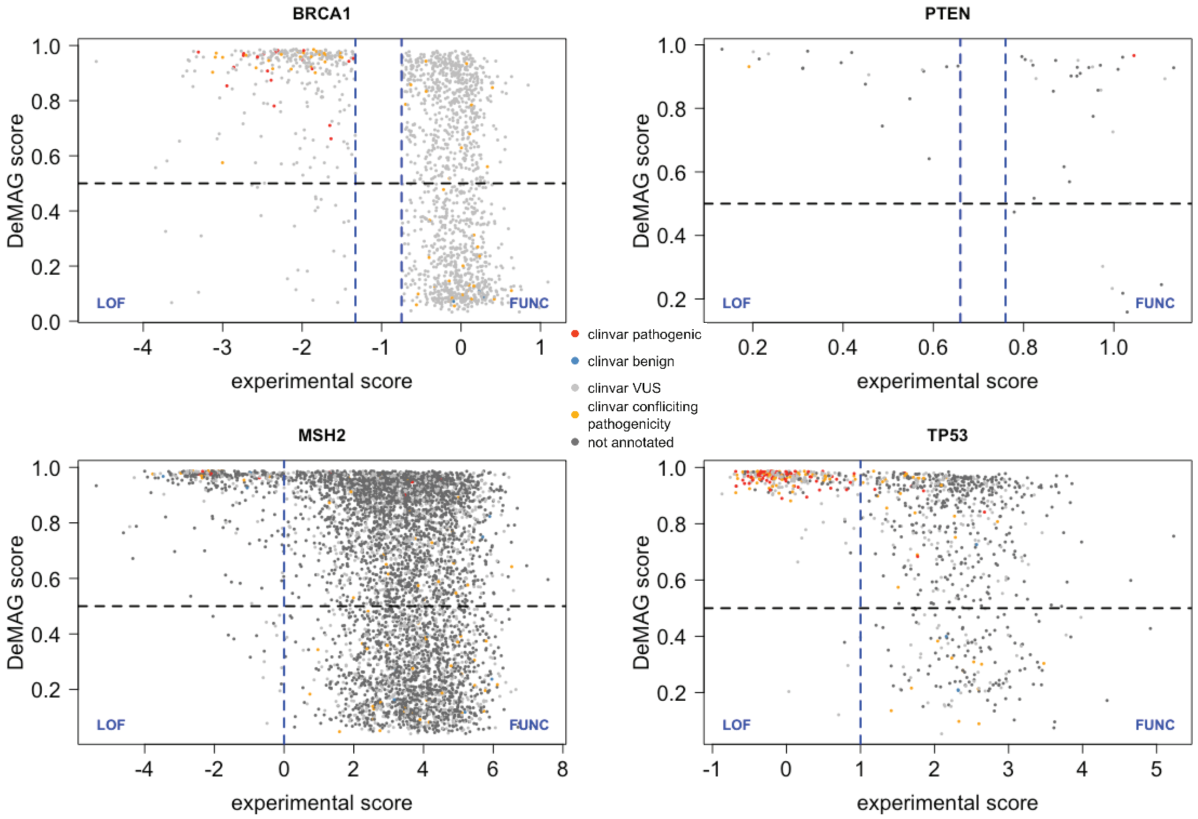

	Review Stars			
**Clinical Significance**	**0**	**1**	**2**	**3**
Benign	0	6	0	21
Pathogenic	1	9	8	48
VUS	11	758	701	10
Conflicting Pathogenicity	0	77	0	0
Not annotated in ClinVar		3562		

VEPs	Sensitivity	Specificity	Accuracy	MCC	AUC	Variants Predicted (n = 5075)	VEP's coverage
DeMAG	0.95	0.38	0.42	0.17	0.86		1
SIFT4G	1	0.04	0.1	0.05	0.82	5075	1
DeMAG	0.95	0.38	0.42	0.17	0.86		1
REVEL	0.98	0.21	0.26	0.12	0.85	5075	1
DeMAG	0.95	0.38	0.42	0.17	0.86		1
DEOGEN2	0.98	0.26	0.31	0.14	0.86	5075	1
DeMAG	0.95	0.38	0.42	0.17	0.86		1
PolyPhen2	0.92	0.49	0.52	0.21	0.8	5075	1
DeMAG	0.95	0.38	0.42	0.17	0.86		1
VEST4	0.98	0.24	0.29	0.13	0.82	5075	1
DeMAG	0.95	0.38	0.42	0.17	0.86		1
M-CAP	0.99	0.06	0.12	0.05	0.8	5075	1
DeMAG	0.96	0.43	0.49	0.24	0.88		1
EVE	0.95	0.68	0.7[1]	0.38	0.89	2909	0.57

[1]The accuracy is 45% if misclassified variants based on EVE's Uncertain class are included.

	Review Stars			
**Clinical Significance**	**0**	**1**	**2**	**3**
Benign	0	4	0	5
Pathogenic	33	45	60	16
VUS	2	201	111	10
Conflicting Pathogenicity	0	96	0	0
Not annotated in ClinVar		539		

VEPs	Sensitivity	Specificity	Accuracy	MCC	AUC	Variants Predicted (n = 1017)	VEP's coverage
DeMAG	0.98	0.22	0.45	0.26	0.86		1
SIFT4G	1	0.03	0.32	0.09	0.81	1017	1
DeMAG	0.98	0.22	0.45	0.26	0.86		1
REVEL	0.99	0.19	0.43	0.24	0.82	1017	1
DeMAG	0.98	0.22	0.45	0.26	0.86		1
DEOGEN2	0.98	0.3	0.5	0.31	0.85	1017	1
DeMAG	0.98	0.21	0.43	0.24	0.87		1
PolyPhen2	0.97	0.33	0.52	0.32	0.78	851	0.84
DeMAG	0.98	0.22	0.45	0.26	0.86		1
VEST4	0.97	0.43	0.59	0.39	0.84	1017	1
DeMAG	0.98	0.22	0.45	0.26	0.86		1
M-CAP	1	0	0.3	0.02	0.72	1017	1
DeMAG	0.99	0.24	0.51	0.3	0.85		1
EVE	0.99	0.36	0.59[1]	0.39	0.86	759	0.75

[1]The accuracy is 44% if misclassified variants based on EVE's Uncertain class are included.

Supplementary Figure 4. Most ClinVar VUSs are predicted as functional variants by DMS data.

DeMAG predictions and functional predictions were compared for four DMS datasets *BRCA1*[6] (top-left), *PTEN*[7] (top-right), *MSH2*[8] (bottom-left), and *TP53*[9] (bottom-right).
On the x-axis the experimental functional score is binned with dashed blue lines in LoF (loss of function), and FUNC (functional) categories, corresponding to the thresholds defined by the authors.
For *BRCA1*, we excluded variants in the intermediate category. We evaluated 1268 (80%) FUNC and 319 (20%) LoF variants.
For *PTEN*, we excluded variants in intermediate categories and variants with standard deviation greater than 10% among the 8 replicas available. We evaluated 34 (64%) FUNC and 19 (36%) LoF variants.
For *MSH2*, the total number of variants analyzed was 5075: 4737 (93%) FUNC and 338 (7%) LoF variants.
For *TP53*, the total number of variants analyzed was 1017 variants: 714 (70%) FUNC and 303 (30%) LOF.
The black dashed line separates DeMAG's predicted pathogenic variants (upper part) from the benign (lower part) ones. The variants are coloured based on their ClinVar annotations.
In each quadrant, the first table shows the ClinVar review status and clinical significance for the DMS variants: most variants are labeled as VUSs. The lower table shows different comparison metrics for the 4 DMS testing sets.
DeMAG has the highest MCC coefficient and the best balance between specificity and sensitivity for *BRCA1*, *MSH2* and *TP53*. For *PTEN*, DeMAG has the highest MCC coefficient but the performance is very low for the functional class (specificity).

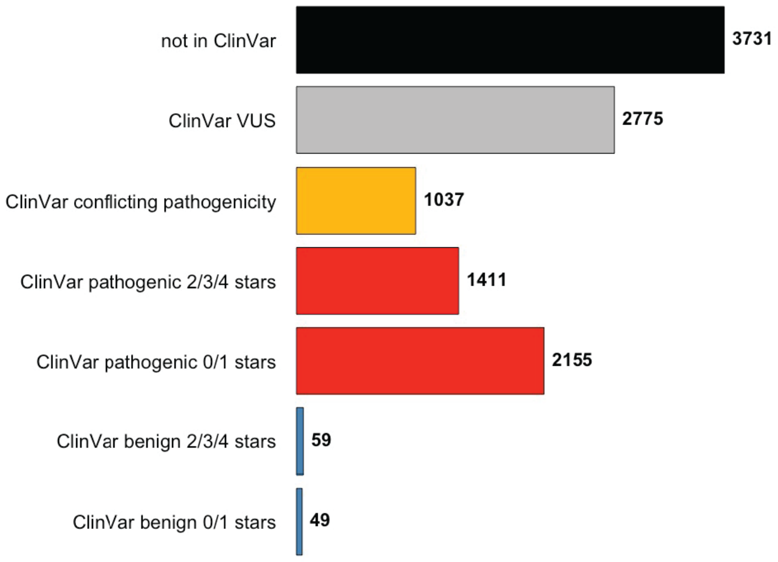

Supplementary Figure 5. Most HGMD variants have a ClinVar label.

The overlap between ClinVar and HGMD databases. Most of HGMD (disease mutation) DM variants have a ClinVar label. Only ca. 30% of variants are in the HGMD database only (black bar). Among the HGMD variants, we mostly found VUSs and low-quality pathogenic variants (0 or 1 review status star). Only 1% of HGMD variants are annotated as benign in ClinVar.

a

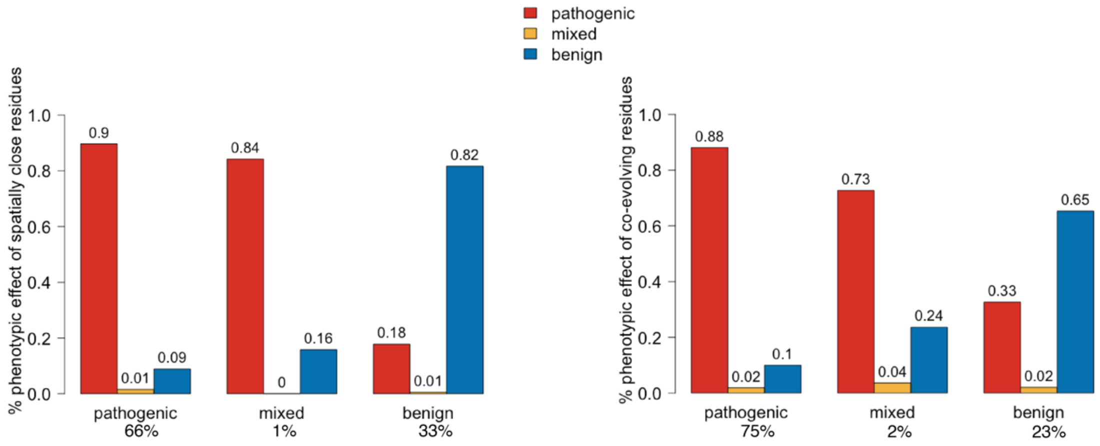

b

Å distance (c-alpha atoms)	AUC	Accuracy	Specificity	Sensitivity	Training set coverage
5	0.78(0.06)	0.79(0.06)	0.36(0.16)	0.92(0.02)	17.9%
6	0.81(0.01)	0.86(0.01)	0.57(0.09)	0.93(0.01)	54.8%
7	0.84(0.02)	0.84(0.03)	0.65(0.08)	0.9(0.02)	67.3%
8	0.86(0.03)	0.84(0.03)	0.62(0.1)	0.91(0.01)	72.6%
9	0.85(0.02)	0.82(0.02)	0.62(0.07)	0.9(0.03)	78.6%
10	0.83(0.01)	0.79(0.03)	0.62(0.02)	0.89(0.02)	82.7%
11	0.88(0.03)	0.84(0.03)	0.73(0.07)	0.88(0.03)	86.4%

Coupling probability	AUC	Accuracy	Specificity	Sensitivity	Training set coverage
0.1	0.84(0.04)	0.84(0.02)	0.61(0.11)	0.92(0.02)	60.1%
0.2	0.85(0.04)	0.83(0.03)	0.6(0.1)	0.92(0.03)	57%
0.3	0.85(0.04)	0.87(0.02)	0.65(0.13)	0.92(0.03)	52%
0.4	0.84(0.05)	0.88(0.02)	0.66(0.12)	0.92(0.02)	46.5%
0.5	0.83(0.05)	0.86(0.03)	0.64(0.11)	0.92(0.02)	40.8%
0.6	0.84(0.05)	0.85(0.03)	0.7(0.09)	0.88(0.03)	35.2%
0.7	0.83(0.05)	0.86(0.03)	0.67(0.1)	0.9(0.03)	30.4%
0.8	0.85(0.03)	0.86(0.02)	0.66(0.1)	0.92(0.01)	24.8%
0.9	0.82(0.04)	0.84(0.04)	0.68(0.1)	0.88(0.05)	18.8%

c

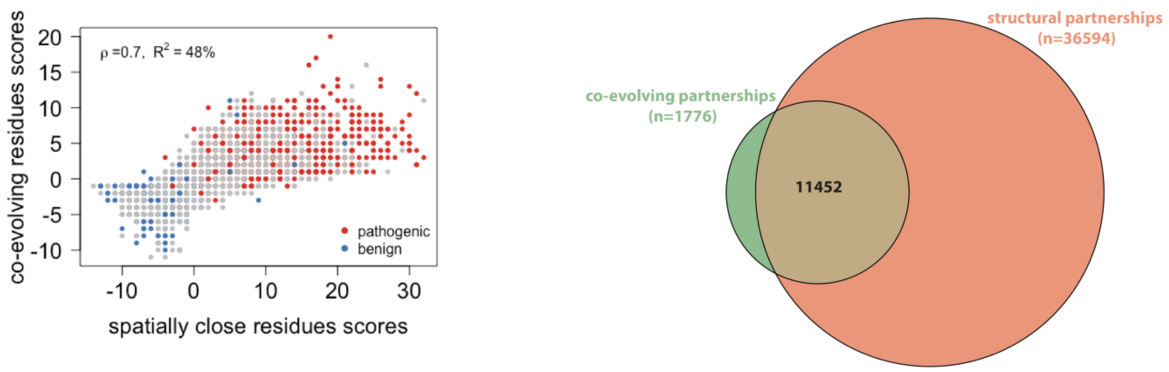

Supplementary Figure 6. Spatially close and co-evolving residues are enriched for the same phenotypic effect.

a) On the left, the phenotypic effect (i.e., training set label) of spatially close residues positions in the training set: pathogenic (benign) residue positions have 90% (82%) pathogenic (benign) partners. Mixed (residues positions associated with both benign and pathogenic variants) positions have mainly (84%) pathogenic partners. Percentages below the x-axis indicate the frequencies of residues positions with spatially close residues for each clinical label in the training set.

On the right, the phenotypic effect of co-evolving residues positions in the training set: pathogenic (benign) residue positions have 88% (65%) pathogenic (benign) partners. Mixed (residues positions associated with both benign and pathogenic variants) positions have mainly (73%) pathogenic partners. Percentages below the x-axis indicate the frequencies of residues positions with co-evolving partners for each clinical label in the training set.

b) The left table shows the performance of the mixture discriminant analysis for different distance cutoffs (see Methods). The right table shows the performance of the mixture discriminant analysis for choosing the best probability cutoff to consider a pair of residues as co-evolving (see Methods).
c) The left plot shows that the correlation between the residue score for the same co-evolving and spatially close residues is high (rho(ρ) 70%, Pearson's product-moment correlation coefficient, greater as alternative hypothesis, p value < 2.2e-16) as well as the variance explained (R^2 48%). The right Venn diagram shows the overlap between co-evolving and structural partnerships: 24% of spatially close residues overlap with co-evolving positions, while 87% of the latter overlap with structural partnerships.

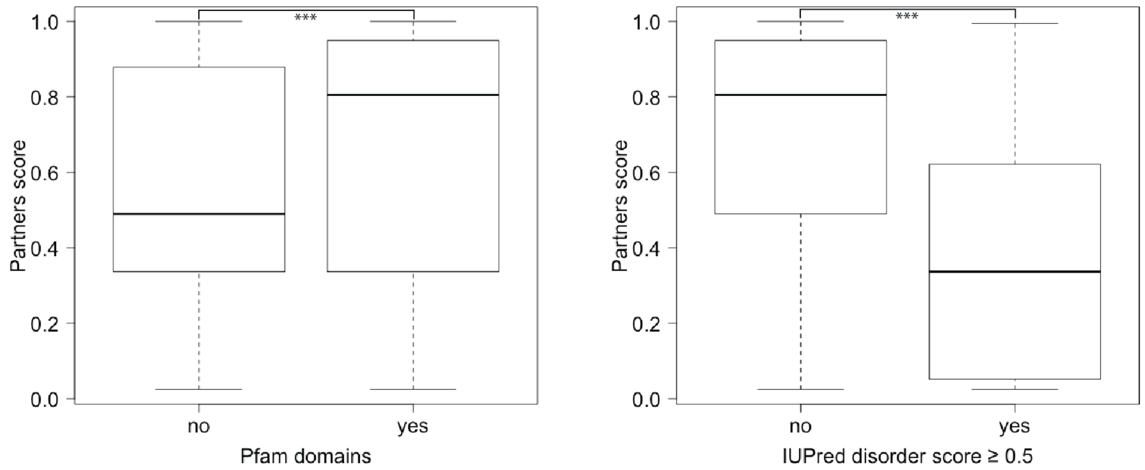

Supplementary Figure 7. Partners score is significantly higher in Pfam domains and in ordered regions.
The boxplot shows that the partners score is significantly higher in Pfam domains and in ordered regions according to IUPred disorder score (disorder score < 0.5) (T-test and Wilcoxon rank test with "greater" as alternative hypothesis with p value < 2.2e-16). The whiskers of the boxplots range correspond to ± 1.5 times the IQR (inter quantile range). The lower (upper) bound of the box of the boxplot corresponds to the 25th (75th) percentile. The center is the median.

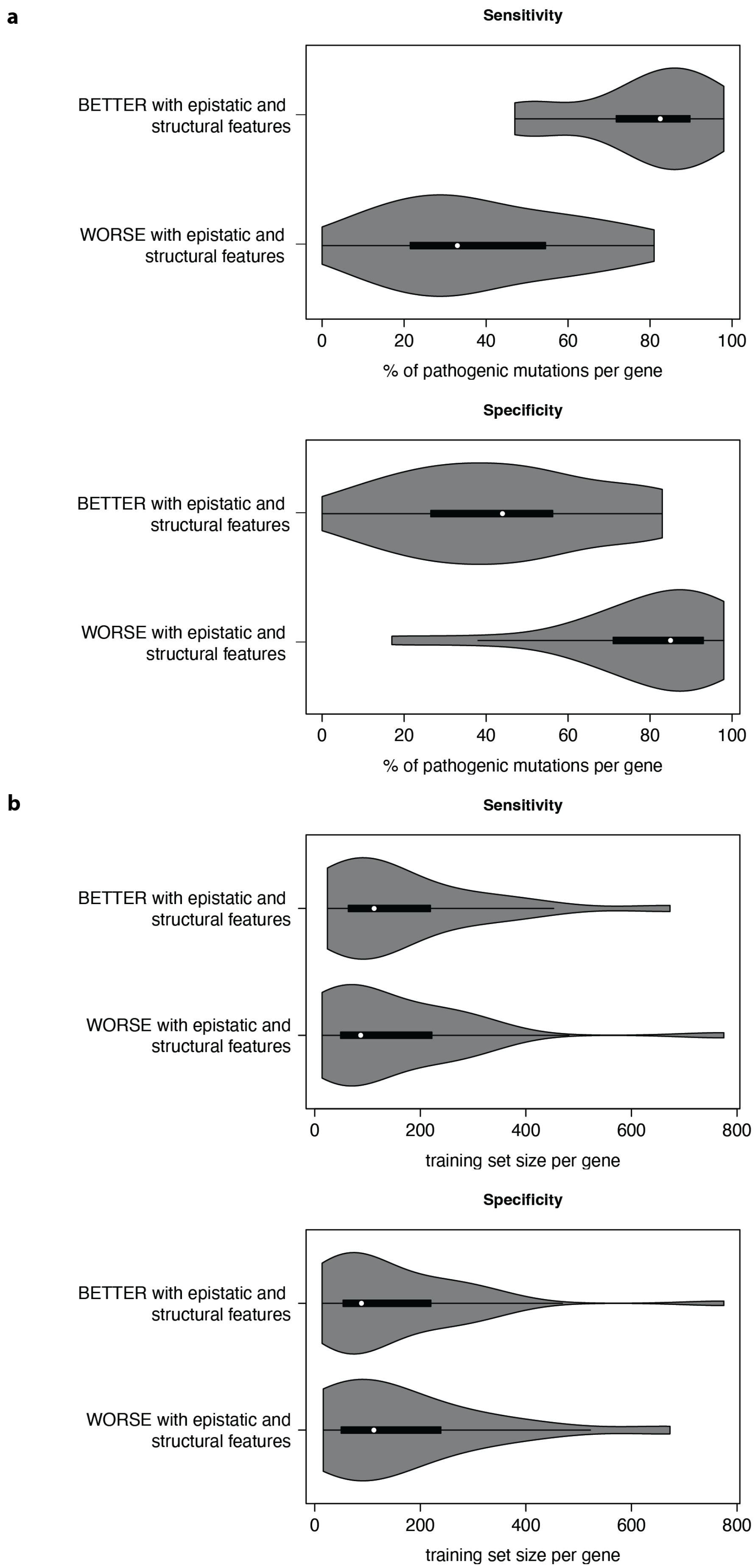

a
Sensitivity
BETTER with epistatic and structural features
WORSE with epistatic and structural features
0 20 40 60 80 100
% of pathogenic mutations per gene
Specificity
BETTER with epistatic and structural features
WORSE with epistatic and structural features
0 20 40 60 80 100
% of pathogenic mutations per gene
b
Sensitivity
BETTER with epistatic and structural features
WORSE with epistatic and structural features
0 200 400 600 800
training set size per gene
Specificity
BETTER with epistatic and structural features
WORSE with epistatic and structural features
0 200 400 600 800
training set size per gene

Supplementary Figure 8. Epistatic and structural features increase sensitivity for "pathogenic" genes and specificity for "benign" genes.

a) The violin plots show the distribution of the ratio of pathogenic mutations for genes whose variants are predicted with higher and lower performance with epistatic and structural features (BETTER and WORSE with epistatic and structural features respectively in the plot). The upper plot (Sensitivity) is based on n=26 and n=31 genes for the BETTER and WORSE with epistatic and structural features groups). The lower plot (Specificity) is based n=36 and n=21 genes for the BETTER and WORSE with epistatic and structural features groups). The whiskers of the boxplot go from minimum to maximum. Sensitivity is higher with epistatic and structural features for pathogenic genes, genes with high content (70%) of pathogenic variants, and worse (lower) for benign genes, genes with low content (30%) of pathogenic variants (Wilcoxon rank sum test, with greater as alternative hypothesis, p value 2.43e-08). Below, the plot shows the same as above with respect to the specificity. In this case, the specificity is higher for benign and lower for pathogenic genes (Wilcoxon rank sum test, with lower as alternative hypothesis, p value 9.431e-07).
b) The description of the violin plots and the groups' size is the same as descried in a). There is no correlation between the size of the training set per gene and the effect of the epistatic and structural features either in terms of better specificity (Wilcoxon rank sum test, with two sided as alternative hypothesis, p value 0.7912) or sensitivity (Wilcoxon rank sum test, with two sided as alternative hypothesis, p value 0.4231).

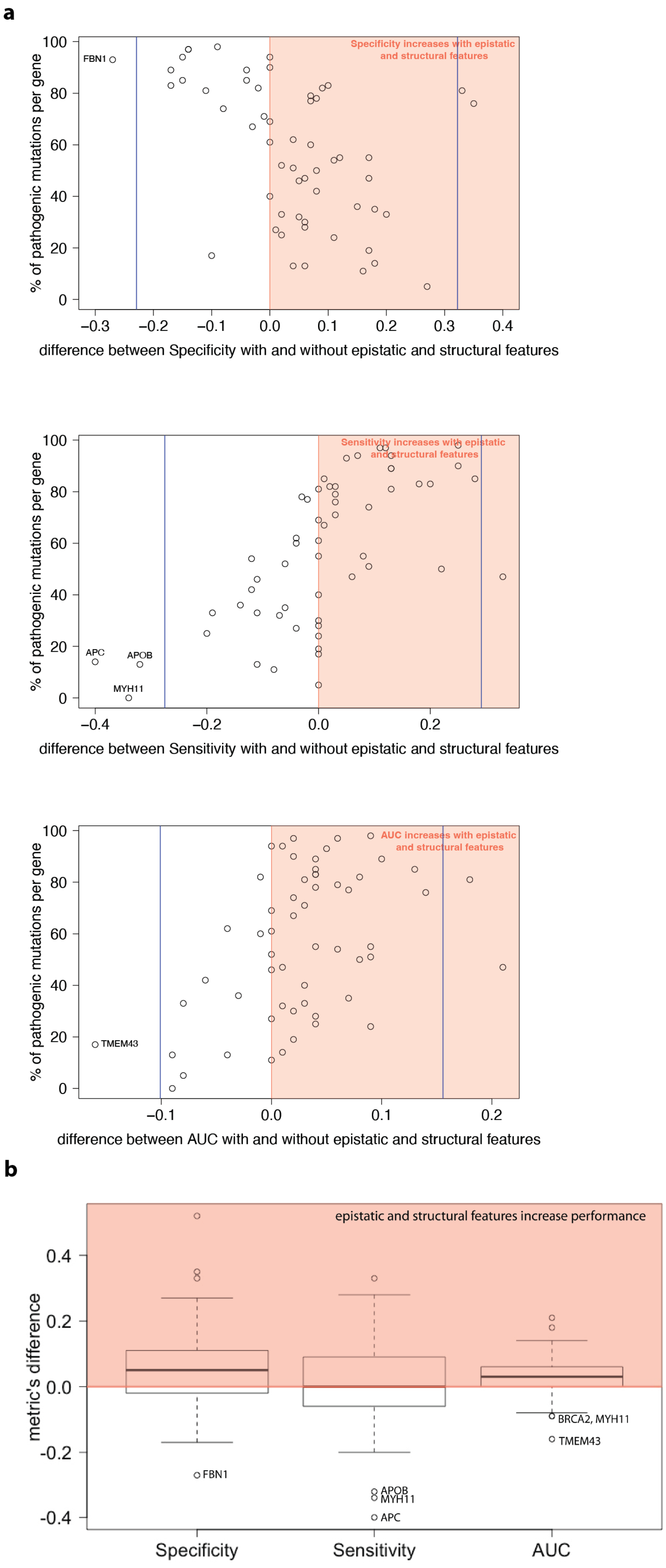
a
100
80
60
40
20
0
% of pathogenic mutations per gene
FBN1
Specificity increases with epistatic and structural features
−0.3
−0.2
−0.1
0.0
0.1
0.2
0.3
0.4
difference between Specificity with and without epistatic and structural features
Sensitivity increases with epistatic and structural features
APC
APOB
MYH11
−0.4
−0.2
0.0
0.2
difference between Sensitivity with and without epistatic and structural features
AUC increases with epistatic and structural features
TMEM43
−0.1
0.0
0.1
0.2
difference between AUC with and without epistatic and structural features
b
epistatic and structural features increase performance
metric's difference
0.4
0.2
0.0
-0.2
-0.4
FBN1
APOB
MYH11
APC
BRCA2, MYH11
TMEM43
Specificity
Sensitivity
AUC

Supplementary Figure. 9. Most genes benefit from the epistatic and structural features.

a) Each plot shows on the x-axis the difference of the performance of the correspondent metric (e.g., Specificity) between DeMAG trained with and without epistatic and structural features and on the y-axis the proportion of pathogenic mutations per each gene, as in our training set. The black lines represent one standard deviation from the mean, while the blue lines two standard deviations. The first plot shows a negative correlation (-54%, Pearson's product-moment correlation coefficient, lower as alternative hypothesis, p value 6.679e-06) between specificity and the ratio of pathogenic mutations. There is instead a positive correlation both with the sensitivity (66%, Pearson's product-moment correlation coefficient, greater as alternative hypothesis, p value 1.546e-08) and the AUC (46%, Pearson's product-moment correlation coefficient, greater as alternative hypothesis, p value 0.0001588) with the ratio of pathogenic mutations.
b) The three boxplots represent the difference of the performance of the correspondent metric (e.g., Specificity) between DeMAG trained with and without epistatic and structural features. Each boxplot is based on n=57 genes, we excluded two genes that have only pathogenic or benign variants because either specificity or sensitivity calculations would have not made sense. The whiskers of the boxplots range correspond to ± 1.5 times the IQR (inter quantile range). The lower (upper) bound of the box of the boxplot corresponds to the 25th (75th) percentile. A positive difference indicates that epistatic and structural features increase performance. Except for sensitivity, most genes benefit from the epistatic and structural features.

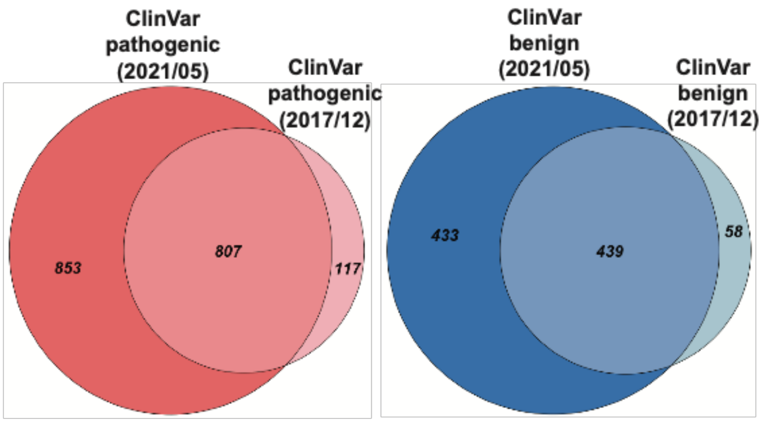

Supplementary Figure 10. The ClinVar test set contains 853 pathogenic and 433 benign variants.

The intersection between two versions of the ClinVar database: from May 2021 and December 2017. The intersection of the two sets is plotted for the pathogenic (left) and benign (right) variants. 853 pathogenic and 433 benign variants were added between 12/2017 to 05/2021.

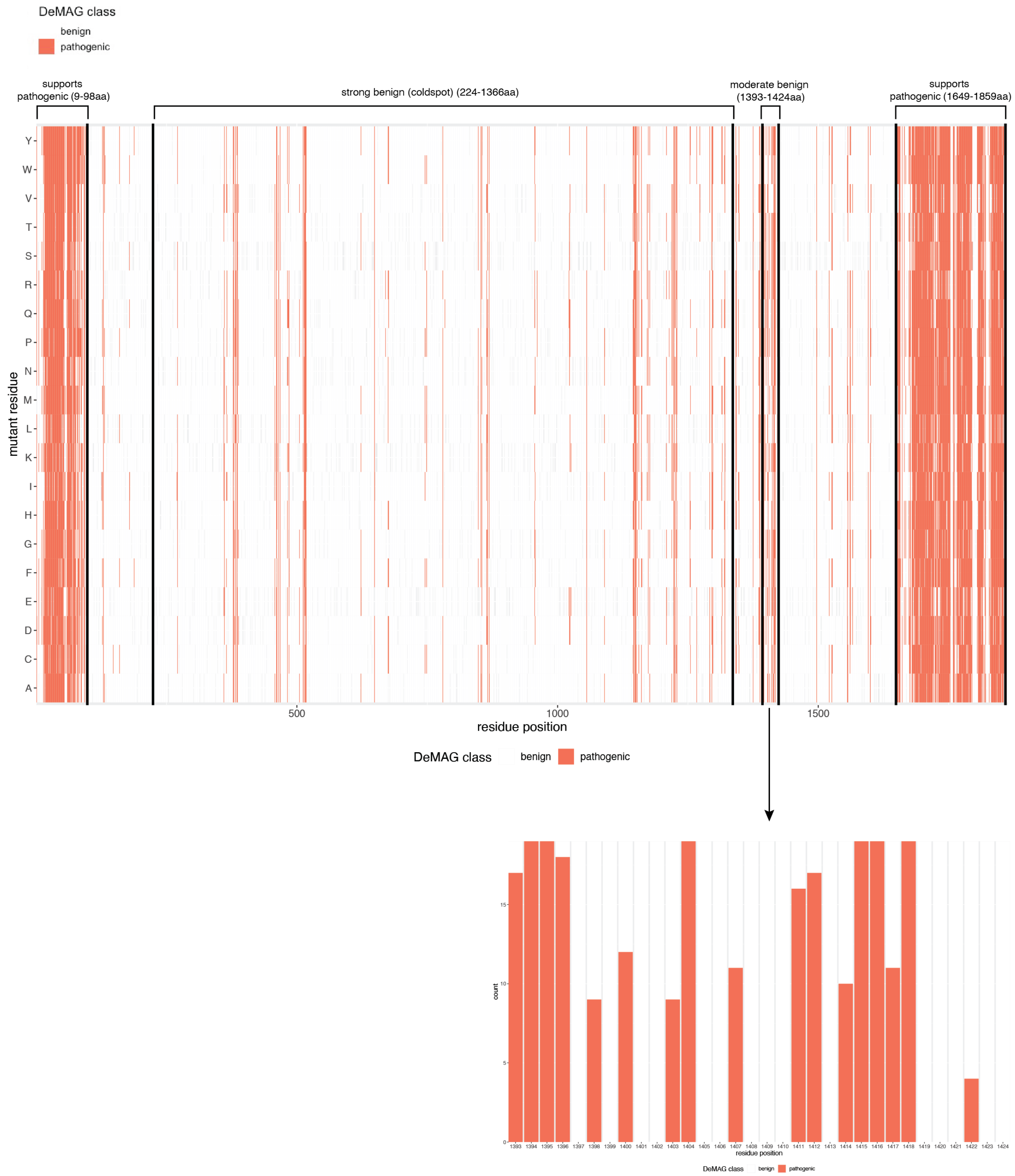

Supplementary Figure 11. DeMAG correctly identifies pathogenic and benign regions (coldspot) in BRCA1.

DeMAG variants classification for all possible substitutions in BRCA1. The light red (white) color identifies variants classified as pathogenic (benign). The gray bars in the plot correspond to the wild-type substitution which is obviously not considered. The black lines indicate regions that have been reclassified[39] e.g., region 224-1366 is considered a coldspot, thus recoded as 'strong benign'. The region from residue 1393 to 1424 (zoomed in the lower plot) has been reclassified as 'moderate benign'

while DeMAG predicts at least 45% of amino acids substitutions as pathogenic. On the x-axis the residue positions and on the y-axis the count of all possible residue substitutions predicted as pathogenic by DeMAG.

a

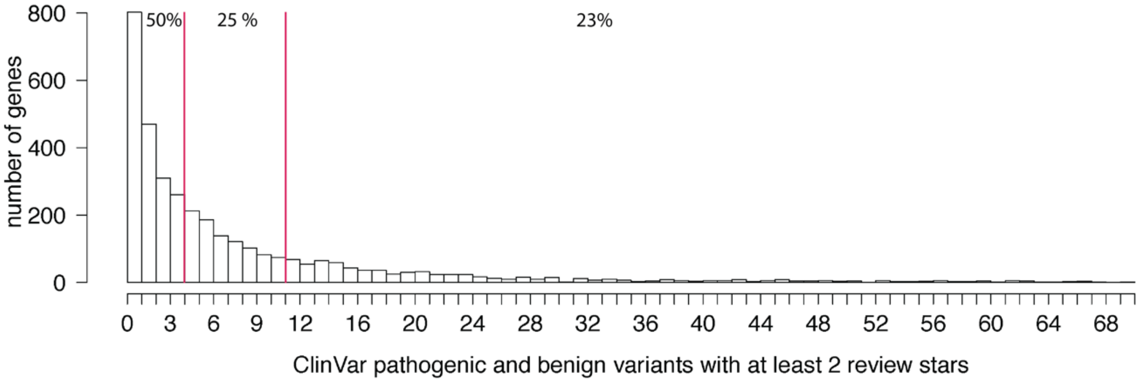

b

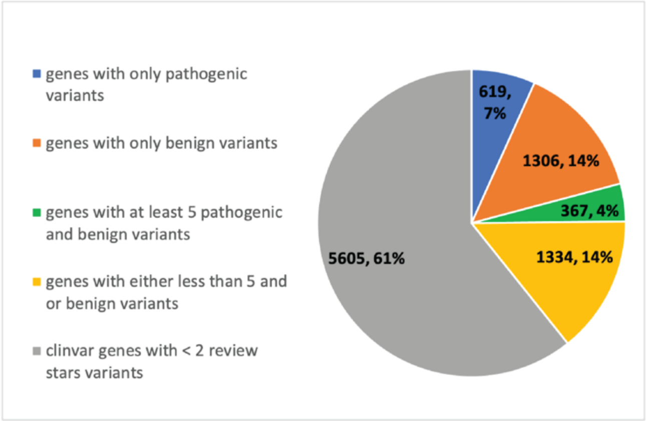

Supplementary Figure 12. Abundant, high-quality and well-balanced data only available for few ClinVar genes.

a) The histogram shows the distribution of high-quality (review status stars ≥ 2) variants across ClinVar genes: 75% of genes have less than 11 variants, 23% have between 11 and 70 variants and the last 2% of genes (not shown in the plot) have between 70 and ~800 variants.

b) The pie chart shows genes distribution based on their associated ClinVar variants. The grey part indicates genes with less than 2 review status stars while the remaining 39% includes genes associated with high-quality variants. Among those, only 4% of genes have at least 5 benign and pathogenic variants.

a

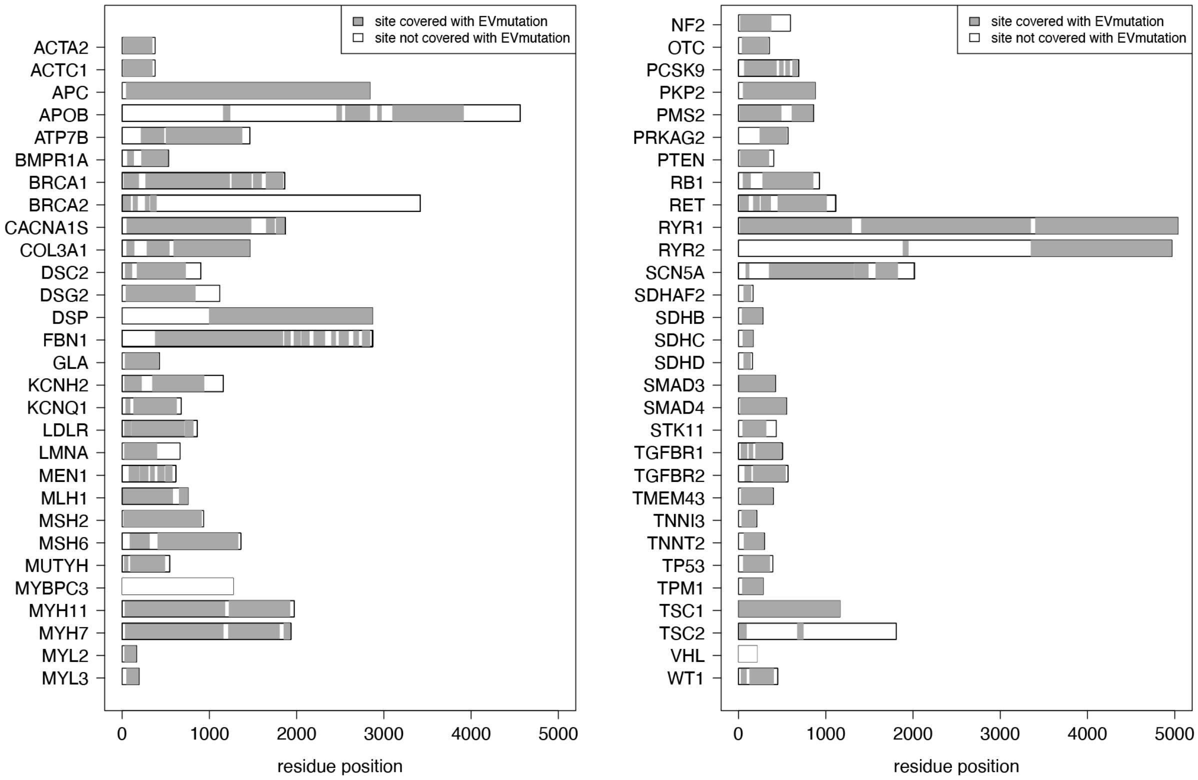
site covered with EVmutation
site not covered with EVmutation
ACTA2
ACTC1
APC
APOB
ATP7B
BMPR1A
BRCA1
BRCA2
CACNA1S
COL3A1
DSC2
DSG2
DSP
FBN1
GLA
KCNH2
KCNQ1
LDLR
LMNA
MEN1
MLH1
MSH2
MSH6
MUTYH
MYBPC3
MYH11
MYH7
MYL2
MYL3
NF2
OTC
PCSK9
PKP2
PMS2
PRKAG2
PTEN
RB1
RET
RYR1
RYR2
SCN5A
SDHAF2
SDHB
SDHC
SDHD
SMAD3
SMAD4
STK11
TGFBR1
TGFBR2
TMEM43
TNNI3
TNNT2
TP53
TPM1
TSC1
TSC2
VHL
WT1
0
1000
2000
3000
4000
5000
residue position

b

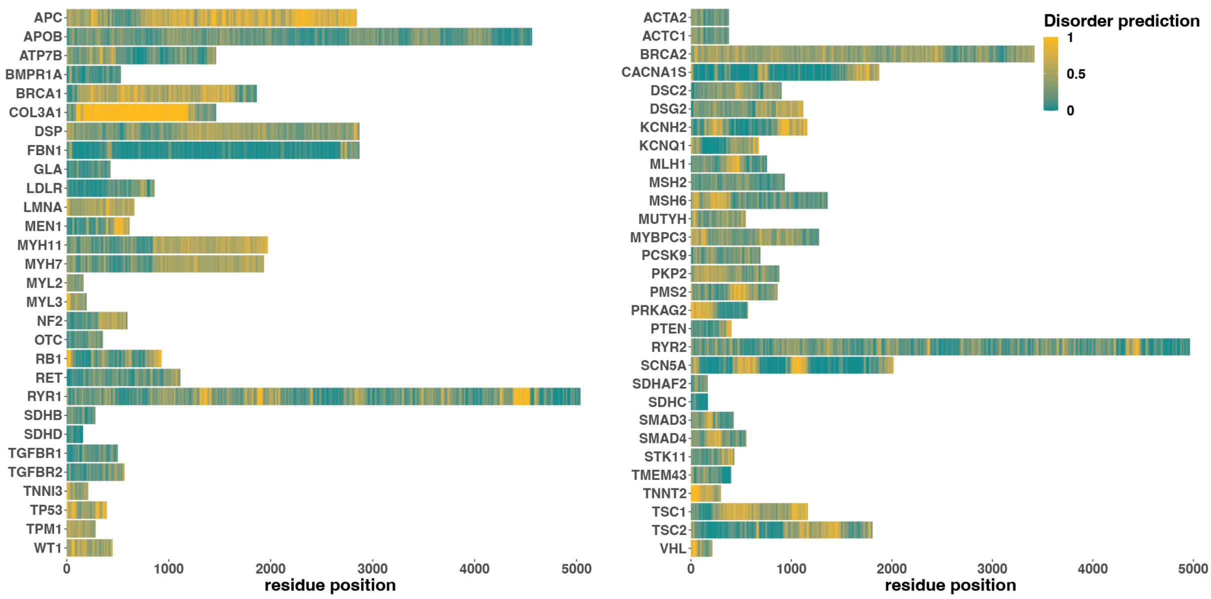
Disorder prediction
1
0.5
0
APC
APOB
ATP7B
BMPR1A
BRCA1
COL3A1
DSP
FBN1
GLA
LDLR
LMNA
MEN1
MYH11
MYH7
MYL2
MYL3
NF2
OTC
RB1
RET
RYR1
SDHB
SDHD
TGFBR1
TGFBR2
TNNI3
TP53
TPM1
WT1
ACTA2
ACTC1
BRCA2
CACNA1S
DSC2
DSG2
KCNH2
KCNQ1
MLH1
MSH2
MSH6
MUTYH
MYBPC3
PCSK9
PKP2
PMS2
PRKAG2
PTEN
RYR2
SCN5A
SDHAF2
SDHC
SMAD3
SMAD4
STK11
TMEM43
TNNT2
TSC1
TSC2
VHL
0
1000
2000
3000
4000
5000
residue position

Supplementary Figure 13. EVmutation covers 73% of ACMG SF sites and 23% of residues are disordered.

a) EVmutation coverage for the ACMG SF genes. The coverage is 64% for ACMG SF sites and is 73% for the training set.
b) The disorder score calculated by IUPred2A for the 59 ACMG SF genes. 23% of residues have a disorder score greater than 0.5.

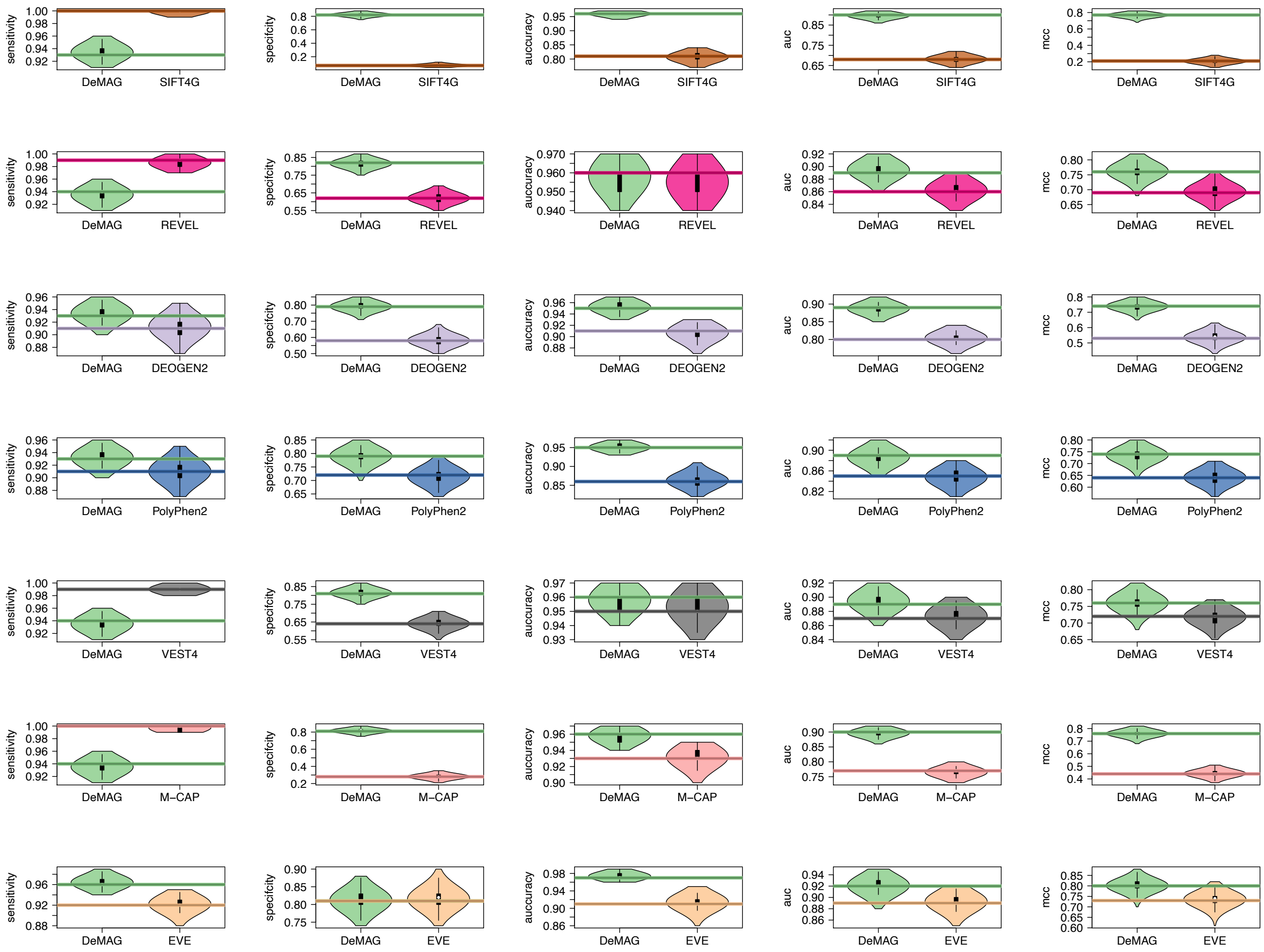

Supplementary Figure 14. Bootstrap confidence intervals for ClinVar testing set.

The violin plots are organized in a 7 by 5 matrix. The rows correspond to 7 different predictors we benchmarked against (e.g., REVEL, SIFT4G) and the columns represent 5 different performance metrics (e.g., sensitivity, specificity). Each violin plot shows the distribution of the respective metric according to 1000 bootstrap samples. The horizontal line shows the respective performance metric as reported in Table 1. The boxplot within the violin plot has a white dot corresponding to the mean of the distribution and the whiskers extend to 1.5 the interquantile range. The comparison with EVE is only shown when uncertain class variants are excluded.

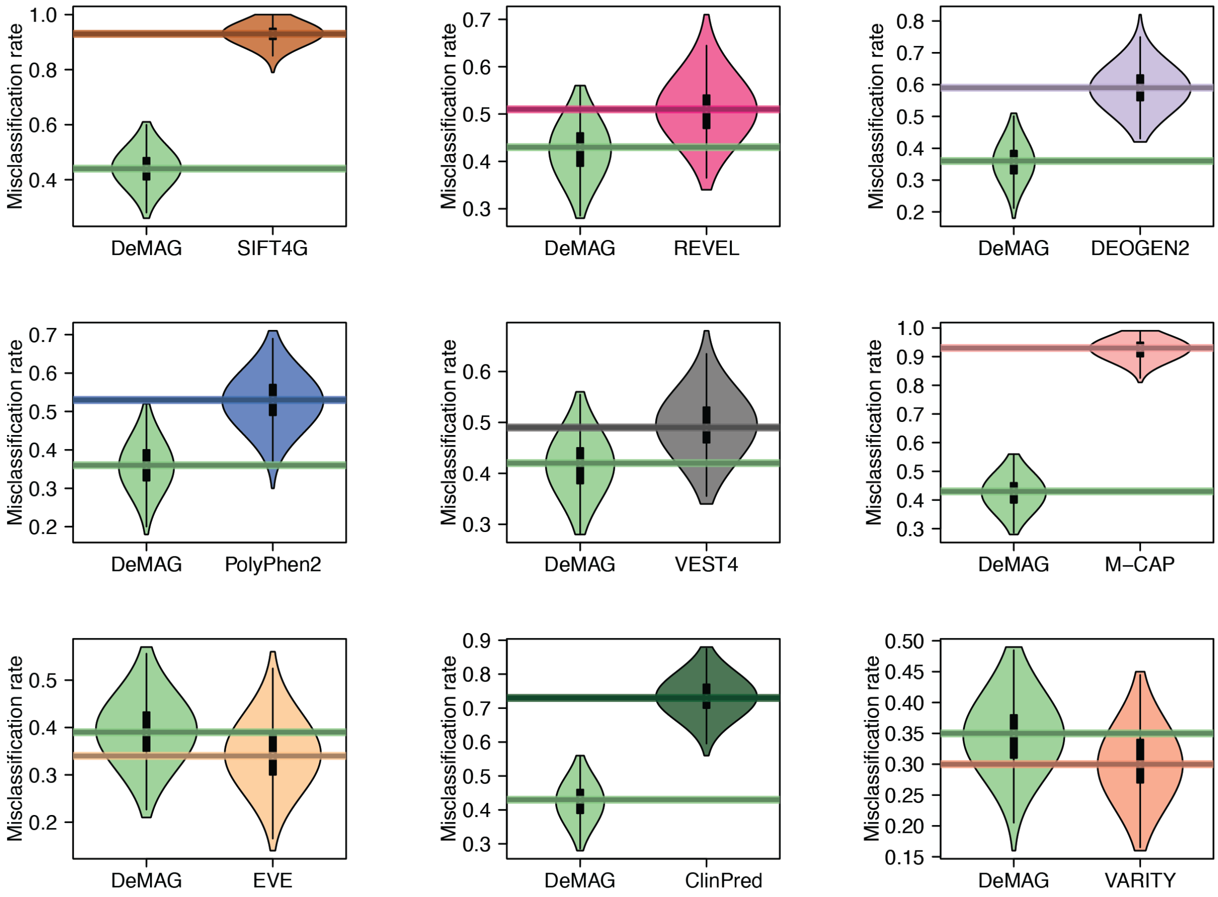

Supplementary Figure 15. Bootstrap confidence intervals for the Estonian variants testing set.

The violin plots show the distribution of the misclassification rate according to 1000 bootstrap samples for the 9 predictors we benchmarked against. The horizontal line shows the respective performance metric as reported in Table 2. The boxplot within the violin plot has a white dot corresponding to the mean of the distribution and the whiskers extend to 1.5 the interquantile range. The comparison with EVE is only shown when uncertain class variants are excluded.

Supplementary Tables

a

Features	AUC	Accuracy	Sensitivity	Specificity	coverage	PPV	NPV
Partners score	0.87	0.83	0.84	0.76	88.2%	0.88	0.71
Nres	0.84	0.8	0.78	0.72	100%	0.85	0.7
EVmutation	0.84	0.82	0.8	0.67	74.3%	0.83	0.75
Nsubs	0.81	0.77	0.77	0.75	100%	0.82	0.68
BaRE	0.81	0.78	0.77	0.72	100%	0.82	0.68
Score1	0.8	0.77	0.82	0.65	100%	0.79	0.72
dScore	0.79	0.78	0.85	0.63	100%	0.78	0.76
phylop	0.79	0.76	0.81	0.58	100%	0.78	0.72
DistQmin	0.77	0.74	0.77	0.63	57.6%	0.64	0.8
DistPmin	0.76	0.74	0.49	0.85	57.6%	0.55	0.79
NormASA	0.74	0.73	0.8	0.58	99.1%	0.75	0.67
FoldX	0.73	0.73	0.79	0.42	75.1%	0.75	0.65
Score2	0.72	0.73	0.85	0.53	100%	0.73	0.7
gerprs	0.72	0.73	0.85	0.5	100%	0.72	0.69
DistPSNP	0.72	0.77	0.2	0.92	22.8%	0.76	0.77
IUPred2A disorder score	0.71	0.73	0.8	0.53	100%	0.74	0.67
AlphaFold2 plddt	0.71	0.74	0.81	0.54	100%	0.76	0.74
B-fact	0.69	0.73	0.88	0.45	99.1%	0.71	0.72
cdn1	0.66	0.67	0.64	0.58	100%	0.74	0.53
aa1	0.66	0.67	0.7	0.52	100%	0.72	0.52
cdn2	0.64	0.62	0.65	0.51	100%	0.63	0.6
Nstr	0.64	0.68	0.56	0.83	99.7%	0.84	0.65
aa2	0.64	0.64	0.73	0.46	100%	0.66	0.54
SecStr	0.63	0.65	0.58	0.58	100%	0.66	0.52
Nvars	0.62	0.64	0.93	0.3	100%	0.61	0.79
H-bonds	0.61	0.72	0.54	0.64	27.9%	0.79	0.36
dProp	0.59	0.61	0.47	0.61	99.1%	0.67	0.44
PfamHit	0.59	0.62	0.76	0.41	100%	0.64	0.52
site	0.57	0.56	0.15	0.99	100%	0.81	0.5
frame	0.56	0.56	0.6	0.5	100%	0.58	0.52
CpG	0.56	0.6	0.44	0.64	100%	0.81	0.53
trv	0.55	0.55	0.48	0.62	100%	0.6	0.51
dgn	0.52	0.55	0.98	0.03	100%	0.54	0.52
region	0.49	0.66	0.87	0.11	100%	0.66	0.36
dVol	0.49	0.62	0.88	0.11	99.1%	0.59	0.32
Nfilt	0.49	0.71	0.97	0	99.7%	0.71	0
Nobs	0.42	0.64	0.96	0.07	100%	0.62	0.56
Nseqs	0.27	0.6	0.57	0.32	100%	0.55	0.28

b

shrinkage	interaction depth	AUC	Accuracy	Sensitivity	Specificity	MCC	PPV	NPV
0.001	1	0.92	0.87	0.87	0.83	0.7	0.89	0.81
0.001	2	0.92	0.87	0.87	0.85	0.71	0.9	0.8
0.001	3	0.92	0.87	0.88	0.82	0.7	0.89	0.82
0.0055	1	0.92	0.87	0.88	0.82	0.7	0.89	0.82
0.0055	2	0.92	0.87	0.88	0.8	0.69	0.88	0.82
0.0055	3	0.92	0.87	0.86	0.84	0.7	0.9	0.8
0.01	1	0.92	0.87	0.89	0.81	0.7	0.88	0.82
0.01	2	0.92	0.86	0.89	0.8	0.69	0.88	0.82
0.01	3	0.92	0.86	0.88	0.81	0.7	0.88	0.81

c

AUC	Accuracy	Sensitivity	Specificity	MCC	PPV	NPV
0.92 (0.01)	0.87 (0.01)	0.87 (0.02)	0.85 (0.03)	0.71 (0.03)	0.90 (0.01)	0.80 (0.03)

Supplementary Table 1. DeMAG model is balanced among different statistics metrics.

a) The results of the feature selection. The first column lists the features names. The following six columns present different statistical metrics, e.g., AUC (ROC-AUC), accuracy. The values are

averages among the 5-fold cross validation (CV) (see Methods). The last column describes the training set coverage for each feature.
b) Statistical metrics for model performance according to 9 combinations of hyperparameters, i.e., shrinkage and interaction depth. The highlighted row in the table marks the selected parameters (see Methods).
c) Performance of DeMAG according to 5-fold cross validation. There are different metrics presented (e.g., sensitivity), with numbers representing mean values and standard deviation in parenthesis.

Feature	Definition	Source
Score1	PSIC score for wild type amino acid residue (aa1)	pph2
Score2	PSIC score for wild type amino acid residue (aa2)	pph2
dScore	difference of PSIC scores for two amino acid residue variants (Score1-Score2)	pph2
DistQmin	minimum distance (sum of branch lengths) along the phylogenetic tree across all substitution types encountered at the substitution position	pph2
phylop	conservation scoring by phyloP (phylogenetic p-values) from the PHAST package for multiple alignments of 99 vertebrate genomes to the human genome	pph2
BaRE	Bayesian Rate Estimator for scoring evolutionary conservation, D.M. Jordan (2015)	pph2
Nres	number of unique residues observed at the substitution position in multiple alignment (without gaps)	pph2
Nsubs	number of residues different from reference residue (aa1) observed at the substitution position in multiple alignment	pph2
EVmutation	A log-odds ratio between the sequence probability of the wild-type and the mutant sequence	T. A. Hopf (2017)
Partners score	residue's posterior probability of pathogenicity given the residue score of its co-evlving and spatially close residues positions	See Methods of the manuscript
NormASA	normalized accessible surface	pph2
IUPred2A disorder score	sequence-based disorder propensity predictor	IUPred2A
AlphaFold2 pLDDT	per-residue confidence metric on a scale from 0-100	AlphaFold2 FAQ

Supplementary Table 2. DeMAG features.
The 'Feature' column indicates the name of the feature, the 'Definition' column a brief description of the feature and the 'Source' column indicates the reference for a detailed features' description: 'pph2' links to http://genetics.bwh.harvard.edu/wiki/pph2/appendix_a. EVmutation can be further explored from the authors publication[10]. For a description of the Partners score see the Methods section of the manuscript. 'IUpred2A' can be better understood from the resources at this link: https://iupred2a.elte.hu/. 'AlphaFold2 FAQ' explains the pLDDT metric and it is available here: https://alphafold.ebi.ac.uk/faq.

a

Features	AUC	Accuracy	Sensitivity	Specificity	coverage	PPV	NPV
Partners score	0.87	0.83	0.82	0.78	88.4%	0.89	0.69
Nres	0.84	0.8	0.77	0.72	100%	0.85	0.69
Nsubs	0.83	0.79	0.79	0.74	100%	0.85	0.68
EVmutation	0.83	0.82	0.8	0.67	74.9%	0.83	0.74
DistPmin	0.81	0.78	0.71	0.76	58%	0.59	0.86
BaRE	0.81	0.77	0.77	0.7	100%	0.81	0.68
Score1	0.79	0.77	0.82	0.65	100%	0.79	0.71
dScore	0.79	0.78	0.85	0.64	100%	0.78	0.74
phylop	0.79	0.76	0.82	0.59	100%	0.78	0.72
DistQmin	0.76	0.74	0.8	0.57	58%	0.62	0.8
NormASA	0.73	0.73	0.82	0.52	93%	0.74	0.67
FoldX	0.73	0.73	0.78	0.44	76.2%	0.75	0.67
Score2	0.72	0.73	0.84	0.53	100%	0.73	0.69
gerprs	0.72	0.73	0.84	0.5	100%	0.72	0.68
DistPSNP	0.72	0.77	0.3	0.9	22.7%	0.52	0.8
IUPred2A disorder score	0.71	0.72	0.83	0.5	100%	0.73	0.66
Nvars	0.71	0.75	0.92	0.48	100%	0.74	0.78
AlphaFold2 plddt	0.7	0.74	0.8	0.54	100%	0.76	0.74
B-fact	0.67	0.72	0.88	0.41	92.7%	0.7	0.7
PfamHit	0.66	0.68	0.75	0.56	100%	0.75	0.57
aa1	0.65	0.67	0.61	0.6	100%	0.74	0.51
cdn1	0.65	0.67	0.67	0.53	100%	0.74	0.51
cdn2	0.63	0.62	0.57	0.59	100%	0.63	0.58
Nstr	0.63	0.68	0.6	0.78	93.5%	0.84	0.67
aa2	0.63	0.64	0.72	0.46	100%	0.66	0.53
H-bonds	0.59	0.72	0.48	0.67	25.7%	0.76	0.39
dProp	0.59	0.61	0.7	0.36	93%	0.64	0.44
site	0.58	0.55	0.17	0.98	100%	0.78	0.5
SecStr	0.57	0.64	0.58	0.5	100%	0.65	0.44
trv	0.56	0.55	0.49	0.62	100%	0.58	0.53
CpG	0.56	0.6	0.44	0.64	100%	0.81	0.54
dVol	0.53	0.64	0.55	0.49	93%	0.72	0.34
frame	0.52	0.52	0.49	0.55	100%	0.52	0.51
dgn	0.51	0.59	0.95	0.06	100%	0.58	0.48
Nfilt	0.49	0.71	0.97	0	93.5%	0.71	0
region	0.46	0.62	0.78	0.12	100%	0.59	0.33
Nobs	0.43	0.64	0.92	0.1	100%	0.62	0.36
Nseqs	0.29	0.59	0.58	0.37	100%	0.59	0.28

b

shrinkage	interaction depth	AUC	Accuracy	Sensitivity	Specificity	MCC	PPV	NPV
0.001	1	0.92	0.87	0.86	0.84	0.7	0.9	0.8
0.001	2	0.92	0.87	0.88	0.82	0.7	0.89	0.82
0.001	3	0.92	0.87	0.88	0.83	0.7	0.89	0.81
0.0055	1	0.92	0.87	0.87	0.83	0.7	0.89	0.8
0.0055	2	0.92	0.86	0.88	0.81	0.69	0.88	0.81
0.0055	3	0.92	0.86	0.86	0.83	0.69	0.9	0.8
0.01	1	0.92	0.87	0.87	0.83	0.7	0.89	0.8
0.01	2	0.92	0.86	0.88	0.81	0.69	0.88	0.82
0.01	3	0.92	0.86	0.87	0.82	0.69	0.89	0.8

c

AUC	Accuracy	Sensitivity	Specificity	MCC	PPV	NPV
0.92 (0.01)	0.87 (0.02)	0.86 (0.02)	0.84 (0.03)	0.70 (0.04)	0.90 (0.01)	0.80 (0.04)

Supplementary Table 3. DeMAG (ClinVar) model is balanced among different statistics metrics.

a) The results of the feature selection. The first column lists the features names. The following six columns present different statistical metrics, e.g., AUC (ROC-AUC), accuracy. The values are average among the different 5-fold CV (see Methods). The last column describes the training set coverage for each feature.

b) Different statistical metrics for model performance according to 9 combinations of hyperparameters, i.e., shrinkage and interaction depth. The highlighted row in the table identifies the combination of selected parameters (see Methods).
c) Performance of DeMAG ClinVar according to 5-fold cross validation. There are different metrics presented (e.g., sensitivity), with numbers representing mean values and standard deviation in parenthesis.

VEP	Sensitivity	Specificity	Accuracy	MCC	AUC	Variants Predicted	Variants coverage
DeMAG	0.91	0.85	0.88	0.75	0.95	7911	1
DeMAG$^{a/b}$	0.93/0.92	0.86/0.83	0.91/0.89	0.79/0.74	0.96/0.95	7911	1
EVE$^{a/b}$	0.89/0.81	0.93/0.86	0.90/0.83	0.78/0.63	0.95/0.93	5427/6682	0.69/0.84

[a]If EVE's Uncertain class variants are excluded from the testing set.
[b]If EVE's Uncertain class variants are assigned as if EVE was a random classifier.

Supplementary Table 4. DeMAG generalizes to additional 257 disease associated genes.
The first line shows DeMAG's performance on 257 ClinVar genes with at least 5 benign and 5 pathogenic high-quality (at least 2 review stars) variants. DeMAG has predictions for all 7911 (100%) variants, and reaches 91% sensitivity and 85% specificity. The high performance of DeMAG confirms that it is a generalizable predictor that can be applied to other genes without re-training the model. Comparison with EVE on the 257 genes: the first numbers (superscript a) refer to the performance on 5427 (69%) variants that EVE predicts as either benign or pathogenic (missing or uncertain predictions are excluded). The second numbers (superscript b) refer to the performance on 6682 variants (84%) which include variants that EVE classifies as uncertain but are high-quality ClinVar benign or pathogenic variants. The assignment of those variants to either class was random. In both cases, DeMAG outperforms EVE.

Data	URL	Date
AlphaFold2 v2.1.0 (for ACMG SF v2.0)	https://alphafold.ebi.ac.uk/download	2021.08.12
AlphaFold2 v2.3.0 (for extra 257 genes)	https://alphafold.ebi.ac.uk/download	2022.09.06
EVmutation	https://marks.hms.harvard.edu/evmutation/human_proteins.html	2022.09.22
PrimateAI (DataFileS1.csv)	The user has to create an account at this link (https://basespace.illumina.com/s/yYGFdGih1rXL)	
KRGDB project	Jung et al., (2019)	
3.5KJPNv2 project	https://humandbs.biosciencedbc.jp/files/hum0015/tommo-3.5kjpnv2-20181105-af_snvall-autosome.zip	
NCBI ALFA	https://ftp.ncbi.nih.gov/snp/population_frequency/latest_release/freq.vcf.gz	2020.11.24
HGMD	Licence version 2020.03. HGMD data were available to the authors under a subscription data use agreement which prohibits sharing variant data from HGMD Professional (QIAGEN). Users and developers may not make HGMD data publicly available. (https://www.hgmd.cf.ac.uk/docs/disclaimer.html)	
ClinVar	https://ftp.ncbi.nlm.nih.gov/pub/clinvar/vcf_GRCh37/archive_2.0/2021/clinvar_20210501.vcf.gz	
	https://ftp.ncbi.nlm.nih.gov/pub/clinvar/vcf_GRCh37/archive_2.0/2017/clinvar_20171203.vcf.gz	
	https://ftp.ncbi.nlm.nih.gov/pub/clinvar/vcf_GRCh37/archive_2.0/2022/clinvar_20220812.vcf.gz	
HumOrtho and UniProt HumVar:	http://genetics.bwh.harvard.edu/downloads/demag/training/	
gnomAD	https://storage.googleapis.com/gcp-public-data--gnomad/release/2.1.1/vcf/exomes/gnomad.exomes.r2.1.1.sites.vcf.bgz.	

EVE	https://evemodel.org/download/bulk	2021.12.16
VARITY	http://varity.varianteffect.org/downloads/varity_all_predictions.tar.gz	2022.01.05
ClinPred	http://www.google.com/url?q=http%3A%2F%2Fhubs.hpc.mcgill.ca%2F~alirezai%2FClinPred&sa=D&sntz=1&usg=AOvVaw1vNR4djxVsQQTH1SCym-V6	2022.11.15

Supplementary Table 5. DeMAG training set data sources.
The table describes the data sources used for assembling the training data.

Supplementary References

1. Landrum, M. J. *et al.* ClinVar: public archive of relationships among sequence variation and human phenotype. *Nucleic Acids Res.* **42**, D980–D985 (2014).
2. Kalia, S. S. *et al.* Recommendations for reporting of secondary findings in clinical exome and genome sequencing, 2016 update (ACMG SF v2.0): a policy statement of the American College of Medical Genetics and Genomics. *Genet. Med.* **19**, 249–255 (2017).
3. Wu, Y., Li, R., Sun, S., Weile, J. & Roth, F. P. Improved pathogenicity prediction for rare human missense variants. *Am. J. Hum. Genet.* **108**, 1891–1906 (2021).
4. Amendola, L. M. *et al.* Performance of ACMG-AMP Variant-Interpretation Guidelines among Nine Laboratories in the Clinical Sequencing Exploratory Research Consortium. *Am. J. Hum. Genet.* **98**, 1067–1076 (2016).
5. Richards, S. *et al.* Standards and guidelines for the interpretation of sequence variants: a joint consensus recommendation of the American College of Medical Genetics and Genomics and the Association for Molecular Pathology. *Genet Med* (2015) doi:10.1038/gim.2015.30.
6. Findlay, G. M. *et al.* Accurate classification of BRCA1 variants with saturation genome editing. *Nature* **562**, 217–222 (2018).
7. Matreyek, K. A. *et al.* Multiplex assessment of protein variant abundance by massively parallel sequencing. *Nat. Genet.* **50**, 874–882 (2018).
8. Jia, X. *et al.* Massively parallel functional testing of MSH2 missense variants conferring Lynch syndrome risk. *Am. J. Hum. Genet.* **108**, 163–175 (2021).
9. Kotler, E., Shani, O., Marks, D. S., Oren, M. & Segal, E. A Systematic p53 Mutation Library Links Differential Functional Impact to Cancer Mutation Pattern and Evolutionary Conservation. *Mol Cell.* **71**, 178–190 (2018).
10. Hopf, T. A. *et al.* Mutation effects predicted from sequence co-variation. *Nat. Biotechnol.* **35**, 128–135 (2017).
