# Supplementary material for: DeMAG predicts the effects of variants in clinically actionable genes by integrating structural and evolutionary epistatic features
Source: Nat Commun. 2023 Apr 19;14:2230. doi: 10.1038/s41467-023-37661-z (PMC10115847; doi:10.1038/s41467-023-37661-z)
Supplement: Supplementary file 3 — Description of Additional Supplementary Files [file 41467_2023_37661_MOESM3_ESM.pdf]

Description of Additional Supplementary Files

Supplementary Data 1 (Supplementary_Data_1.xlsx)

There are two excel sheets. The first sheet is called 'ClinVar variants per gene'. The first three columns' names are the same as the previous excel sheet. The list of genes is larger because it includes also genes with less than 5 benign and 5 pathogenic variants. There is a table created with the Pivot Table functionality in Excel to make the pie chart plot. The table is as well copy-pasted for convenience.

The second one is called 'Variants count in the 257 genes' and contains the number of variants that we used for testing DeMAG on extra 257 ClinVar genes (with at least 5 benign and 5 pathogenic variants), based on ClinVar version 2022.08.12. Quality filters are explained in the Methods section of the manuscript.

Columns' names:

- UniProt id: UniProt id of the protein.
- # pathogenic: counts of pathogenic variants for that gene.
- # benign: counts of benign variants for that gene.

Supplementary Data 2 (Supplementary_Data_2.xlsx)

The excel file contains the diseases prevalence values for the phenotype of the ACMG SF v2.0 genes.

Columns' names:

- Disease name and MIM number. Phenotype name according to https://www.ncbi.nlm.nih.gov/clinvar/docs/acmg/.
- Gene via GTR. Gene name
- Gene. Gene name
- Prevalence of the disease. Prevalence values of the disease as reported in Orphanet, MedlinePlus or publications. Multiple values are reported for the sake of completeness.
- Prevalence of the disease (%, most conservative). The prevalence value used to threshold variants Minor Allele Frequency (MAF). See the Methods section for details.
- ICD-10 code. When available we reported the ICD-10 code of the phenotype.
- Inheritance: When available we reported the mode of inheritance of the phenotype.
- Link: Source of the data that was collected to extract disease prevalence values.
- Notes: If necessary specific comments are added for the sake of clarity.
