# Supplementary material for: DeMAG predicts the effects of variants in clinically actionable genes by integrating structural and evolutionary epistatic features
Source: Nat Commun. 2023 Apr 19;14:2230. doi: 10.1038/s41467-023-37661-z (PMC10115847; doi:10.1038/s41467-023-37661-z)
Supplement: Supplementary file 6 — Reporting Summary [file 41467_2023_37661_MOESM6_ESM.pdf]

nature portfolio

Corresponding author(s): Agnes Toth-Petroczy

Last updated by author(s): Feb 27, 2023

Reporting Summary

Nature Portfolio wishes to improve the reproducibility of the work that we publish. This form provides structure for consistency and transparency in reporting. For further information on Nature Portfolio policies, see our Editorial Policies and the Editorial Policy Checklist.

Statistics

For all statistical analyses, confirm that the following items are present in the figure legend, table legend, main text, or Methods section.

n/a	Confirmed	
☐	☒	The exact sample size (*n*) for each experimental group/condition, given as a discrete number and unit of measurement
☒	☐	A statement on whether measurements were taken from distinct samples or whether the same sample was measured repeatedly
☐	☒	The statistical test(s) used AND whether they are one- or two-sided<br>*Only common tests should be described solely by name; describe more complex techniques in the Methods section.*
☐	☒	A description of all covariates tested
☐	☒	A description of any assumptions or corrections, such as tests of normality and adjustment for multiple comparisons
☐	☒	A full description of the statistical parameters including central tendency (e.g. means) or other basic estimates (e.g. regression coefficient) AND variation (e.g. standard deviation) or associated estimates of uncertainty (e.g. confidence intervals)
☐	☒	For null hypothesis testing, the test statistic (e.g. *F*, *t*, *r*) with confidence intervals, effect sizes, degrees of freedom and *P* value noted<br>*Give P values as exact values whenever suitable.*
☒	☐	For Bayesian analysis, information on the choice of priors and Markov chain Monte Carlo settings
☒	☐	For hierarchical and complex designs, identification of the appropriate level for tests and full reporting of outcomes
☒	☐	Estimates of effect sizes (e.g. Cohen's *d*, Pearson's *r*), indicating how they were calculated

Our web collection on statistics for biologists contains articles on many of the points above.

Software and code

Policy information about availability of computer code

Data collection

AlphaFold2: https://alphafold.ebi.ac.uk/download (v2.1.0 for the ACMG SF v2.0 genes and v2.3.0 for the extra 257 genes). EVmutation: downloaded on the 2022.09.22, https://marks.hms.harvard.edu/evmutation/human_proteins.html. Disease prevalence values: https://www.orpha.net, https://medlineplus.gov/. ClinVar VCF file: https://ftp.ncbi.nlm.nih.gov/pub/clinvar/vcf_GRCh37/archive_2.0/2021/clinvar_20210501.vcf.gz, https://ftp.ncbi.nlm.nih.gov/pub/clinvar/vcf_GRCh37/archive_2.0/2017/clinvar_20171203.vcf.gz. PrimateAI data: The user has to create an account at this link (https://basespace.illumina.com/s/yYGFdGih1rXL) to access the data (DataFileS1.csv). KRGDB project: email the author to ask for the data. 3.5KJPNv2 project: https://humandbs.biosciencedbc.jp/files/hum0015/tommo-3.5kjpnv2-20181105-af_snvall-autosome.zip. NCBI ALFA: https://ftp.ncbi.nih.gov/snp/population_frequency/latest_release/freq.vcf.gz. HGMD variants version 2020.03 are license-protected. ClinVar VCF file (extra 257 genes): https://ftp.ncbi.nlm.nih.gov/pub/clinvar/vcf_GRCh37/archive_2.0/2022/clinvar_20220812.vcf.gz. HumOrtho and UniProt HumVar: http://genetics.bwh.harvard.edu/downloads/demag/training/. gnomAD: https://storage.googleapis.com/gcp-public-data--gnomad/release/2.1.1/vcf/exomes/gnomad.exomes.r2.1.1.sites.vcf.bgz. HGMD data were available to the authors under a subscription data use agreement which prohibits sharing variant data from HGMD Professional (QIAGEN). Users and developers may not make HGMD data publicly available. (https://www.hgmd.cf.ac.uk/docs/disclaimer.html). EVE precomputed scores: downloaded on 2021.12.16 https://evemodel.org/download/bulk. VARITY precomputed scores: downloaded on 2022.01.05 (http://varity.varianteffect.org/downloads/varity_all_predictions.tar.gz). ClinPred precomputed scores: downloaded on 2022.11.15 (http://www.google.com/url?q=http%3A%2F%2Fhubs.hpc.mcgill.ca%2F~alirezai%2FClinPred&sa=D&sntz=1&usg=AOvVaw1vNR4djxVsQQTH1SCym-V6).

Data analysis	https://iupred.elte.hu (v2), https://github.com/debbiemarkslab/EVcouplings, https://git.mpi-cbg.de/tothpetroczylab/DeMAG, R packages: gbm v2.1.8, ROCR v1.0.11, arcdiagram 0.1.12, shiny 1.6.0, data.table 1.13.6, NGLVieweR 1.3.1, mclust 5.4.7, caret 6.0.88, mltools 0.3.5, ggplot2 v3.3.5; PolyPhen-2 v2.2.3. and MapSNPs tool from PolyPhen-2 v2.2.3. dbNSFP v4.1a command-line application.

For manuscripts utilizing custom algorithms or software that are central to the research but not yet described in published literature, software must be made available to editors and reviewers. We strongly encourage code deposition in a community repository (e.g. GitHub). See the Nature Portfolio guidelines for submitting code & software for further information.

Data

Policy information about availability of data

All manuscripts must include a data availability statement. This statement should provide the following information, where applicable:

- Accession codes, unique identifiers, or web links for publicly available datasets
- A description of any restrictions on data availability
- For clinical datasets or third party data, please ensure that the statement adheres to our policy

All data generated or analysed during this study is available at demag.org and at this DOI: https://doi.org/21.11101/0000-0007-FB84-9.

Human research participants

Policy information about studies involving human research participants and Sex and Gender in Research.

Reporting on sex and gender	NA
Population characteristics	NA
Recruitment	NA
Ethics oversight	NA

Note that full information on the approval of the study protocol must also be provided in the manuscript.

Field-specific reporting

Please select the one below that is the best fit for your research. If you are not sure, read the appropriate sections before making your selection.

☒ Life sciences ☐ Behavioural & social sciences ☐ Ecological, evolutionary & environmental sciences

For a reference copy of the document with all sections, see nature.com/documents/nr-reporting-summary-flat.pdf

Life sciences study design

All studies must disclose on these points even when the disclosure is negative.

Sample size	NA
Data exclusions	ClinVar VCF file, version 2021.05, we retained variants with a review status of least 2 stars, with either a 'pathogenic' or 'benign' clinical significance (including likely benign and likely pathogenic labels). Variants of conflicting interpretations were excluded as well as variants which had only somatic labels. We used the Human Mutation Gene Database40 (HGMD), version 2020.03, to extract additional pathogenic mutations. We filtered for disease mutations (DMs) and retained variants that were not already annotated in ClinVar.
Replication	NA
Randomization	NA
Blinding	NA

Reporting for specific materials, systems and methods

We require information from authors about some types of materials, experimental systems and methods used in many studies. Here, indicate whether each material, system or method listed is relevant to your study. If you are not sure if a list item applies to your research, read the appropriate section before selecting a response.

Materials & experimental systems

n/a	Involved in the study
☒	☐ Antibodies
☒	☐ Eukaryotic cell lines
☒	☐ Palaeontology and archaeology
☒	☐ Animals and other organisms
☒	☐ Clinical data
☒	☐ Dual use research of concern

Methods

n/a	Involved in the study
☒	☐ ChIP-seq
☒	☐ Flow cytometry
☒	☐ MRI-based neuroimaging
